# Post-traumatic stress reactions in young victims of road traffic accidents

**DOI:** 10.1080/20008198.2017.1351163

**Published:** 2017-09-29

**Authors:** Stella Charitaki, Panagiota Pervanidou, John Tsiantis, George Chrousos, Gerasimos Kolaitis

**Affiliations:** ^a^ Department of Child Psychiatry, Medical School, National and Kapodistrian University of Athens, ‘Aghia Sophia’ Children’s Hospital, Athens, Greece; ^b^ First Department of Paediatrics, Medical School, National and Kapodistrian University of Athens, Athens, Greece

**Keywords:** Traffic accidents, post-traumatic stress disorder, children and adolescents

## Abstract

Many children and adolescents are exposed to traumatic experiences every year. Over 30% of these children develop a clinical syndrome with emotional, behavioural, cognitive, social, and physical symptoms called post-traumatic stress disorder (PTSD). Road traffic accidents (RTAs) are the main cause of morbidity and mortality in ages 0–19 in industrialized countries. In Greece, 30,000 individuals are injured and 2000 die in RTAs every year, 40% of them between 15 and 34 years old. Findings from previous research exhibit that each fatal motor vehicle accident is related to 50 severe injuries. Although distress after the accident can persist for several months and the need of early assessment and treatment has been highlighted, the emotional needs of children involved in RTAs are rarely recognized and children have received hardly any planned intervention (Kramer & Landolt, 2014). In addition, studies on the extent to which the degree of physical injury predicts PTSD development show mixed results (Delahanty, Raimonde, Spoonster, and Cullado, 2003). In these relatively limited studies, contradicting results concerning the relationship between demographic, accident and trauma factors and the psychological symptoms are shown.

In a previous study (Pervanidou et al., 2007), which was conducted over a two-year period, the main aim was to identify and evaluate the severity of PTSD symptoms after a RTA. Furthermore, epidemiological, psychosocial and clinical characteristics related to the disorder were reviewed. The sample consisted of 57 children (39 male, 18 female), aged 7–18, who were admitted to ‘Aghia Sophia’ Children’s General Hospital and other hospitals. Self-reported questionnaires and a semi-structured psychiatric diagnostic interview (K-SADS-PL) were administered. Stress-related hormones were also measured. Assessment took place during the admission to the general hospital, in one month, and six months later. There was a community control group of 30 children/adolescents.

Concerning the type of the accident, 36 (59%) of the young victims were traumatized as pedestrians, while 25 (41%) were passengers. The severity of body injury was mild in 21 (34.4%), moderate in 24 (39.3%) and severe in 16 (26.2%) victims. Results showed that 38.3% of children/adolescents who were involved in a RTA suffered from PTSD one month afterwards, while the percentage is significantly lower (15%) six months after the RTA. PTSD symptoms were experienced by children of all ages, although boys were most likely to be affected, especially those who were severely injured during the accident. The only predictor variable that had a significant positive effect on the development of PTSD (one month after the accident) was current maternal PTSD symptoms. Important findings regarding the consequent short- and/or long-term alterations on the circulating concentrations of stress hormones and adipo-cytokines were established as well (Pervanidou et al., 2007).

Despite increasing attention over the past 20 years, PTSD is a significant public health issue that has not been properly addressed. It is well documented that highly stressful events affect the normal activity of the neurotransmitter norepinephrine and elevate levels of the hormone cortisol. Furthermore, continuous arousal during the course of time may produce difficulty in the hippocampus’s ability to manage in the future highly stressful events. Children and adolescents may respond to a traumatic event differently, depending on the degree of exposure, the age or developmental stage, the effect of the personal impact and the proximity to the actual traumatizing event. In our study, almost one out of three young victims of RTAs was found to suffer from PTSD a month later, confirming previous findings. The child’s perception of the accident was important, with badly injured children being more likely to develop PTSD. Further research could contribute to improved treatment approaches to reduce the devastating personal and societal impact of PTSD.

## References

[CIT0001] Delahanty D. L., Raimonde A. J., Spoonster E., & Cullado M. (2003). Injury severity, prior trauma history, urinary cortisol levels, and acute PTSD in motor vehicle accident victims. *Journal of Anxiety Disorders*, 17, 1–2. 12614659 10.1016/s0887-6185(02)00185-8

[CIT0002] Kramer D. N., & Landolt M. A. (2014). Early psychological intervention in accidentally injured children ages 2–16: A randomized controlled trial. *European Journal of Psychotraumatology*, 5. doi:10.3402/ejpt.v5.24402 PMC4074605 24987498

[CIT0003] Pervanidou P., Kolaitis G., Charitaki S., Lazaropoulou C., Papassotiriou I., Hindmarsh P., … Chrousos G. P. (2007). The natural history of neuroendocrine changes in pediatric posttraumatic stress disorder (PTSD) after motor vehicle accidents: progressive divergence of noradrenaline and cortisol concentrations over time. *Biological Psychiatry*, 62(10), 1095–1102. doi:10.1016/j.biopsych.2007.02.008 17624319

